# Helmet CPAP to Treat Acute Hypoxemic Respiratory Failure in Patients with COVID-19: A Management Strategy Proposal

**DOI:** 10.3390/jcm9041191

**Published:** 2020-04-22

**Authors:** Dejan Radovanovic, Maurizio Rizzi, Stefano Pini, Marina Saad, Davide Alberto Chiumello, Pierachille Santus

**Affiliations:** 1 Department of Biomedical and Clinical Sciences (DIBIC), Division of Respiratory Diseases, Università degli Studi di Milano, Ospedale L. Sacco, ASST Fatebenefratelli-Sacco, Via G.B. Grassi, 74-20157 Milano, Italy; dejan.radovanovic@asst-fbf-sacco.it (D.R.); maurizio.rizzi@asst-fbf-sacco.it (M.R.); stefano.pini@unimi.it (S.P.); marina.saad@icloud.com (M.S.); 2 SC Anestesia e Rianimazione, Ospedale San Paolo-Polo Universitario, ASST Santi Paolo e Carlo, Dipartimento di Scienze della Salute, Università degli Studi di Milano, Via Antonio di Rudinì, 8-20142 Milano, Italy; davide.chiumello@unimi.it; 3 Centro Ricerca Coordinata di Insufficienza Respiratoria, 20123 Milano, Italy

**Keywords:** continuous positive airway pressure, positive end-expiratory pressure, COVID-19, severe acute respiratory syndrome coronavirus 2 (SARS-CoV-2), respiratory failure, helmet, hypoxia, pneumonia

## Abstract

Since the beginning of March 2020, the coronavirus disease 2019 (COVID-19) pandemic has caused more than 13,000 deaths in Europe, almost 54% of which has occurred in Italy. The Italian healthcare system is experiencing a stressful burden, especially in terms of intensive care assistance. In fact, the main clinical manifestation of COVID-19 patients is represented by an acute hypoxic respiratory failure secondary to bilateral pulmonary infiltrates, that in many cases, results in an acute respiratory distress syndrome and requires an invasive ventilator support. A precocious respiratory support with non-invasive ventilation or high flow oxygen should be avoided to limit the droplets’ air-dispersion and the healthcare workers’ contamination. The application of a continuous positive airway pressure (CPAP) by means of a helmet can represent an effective alternative to recruit diseased alveolar units and improve hypoxemia. It can also limit the room contamination, improve comfort for the patients, and allow for better clinical assistance with long-term tolerability. However, the initiation of a CPAP is not free from pitfalls. It requires a careful titration and monitoring to avoid a delayed intubation. Here, we discuss the rationale and some important considerations about timing, criteria, and monitoring requirements for patients with COVID-19 respiratory failure requiring a CPAP treatment.

## 1. Effects of the Severe Acute Respiratory Syndrome Coronavirus 2 (SARS-CoV-2) Pandemic in Italy

The World Health Organization (WHO) has recently declared the SARS-CoV-2 infection-related disease (coronavirus disease 2019; COVID-19) a pandemic. As of 26 March 2020, the WHO reported 250,592 confirmed COVID-19 cases in Europe with 13,950 deaths, almost 54% of which had occurred in Italy [1]. The dynamics of the viral diffusion in Italy, especially in the northern region of Lombardy, lead to a stressful burden on the healthcare system, particularly on the emergency departments and the intensive care units (ICU), with almost 10% of the hospitalized COVID-19 patients with hypoxic respiratory failure (HRF) needing invasive respiratory assistance. To avoid aqueous droplets dispersion during active disease, the use of high flow oxygen and non-invasive mechanical ventilation (NIMV) is generally not recommended in case of the unavailability of negative pressure isolation rooms, high level protective equipment, and adequate monitoring systems [2,3], usually currently lacking in the majority of the medical units involved in the management of COVID-19 patients during the emergency. Moreover, the application of non-invasive ventilation (NIV) in patients with acute respiratory distress syndrome (ARDS) complicating a viral pneumonia has been demonstrated to be unable to change the clinical course of the disease [3]. However, the frequent lack of ICU beds have pushed the authorities to create numerous new respiratory intermediate care units (RICU) and convert to this purpose many general internal medicine units, in order to face the increasing number of patients with severe pneumonia and ARDS-needing respiratory support and monitoring.

## 2. Pathology and Pathophysiology of COVID-19 Pulmonary Damage

The pathophysiological consequence of the bilateral ground glass opacities and the parenchymal consolidations, as seen in COVID-19 pneumonia, is represented by a substantial intrapulmonary shunt likely coupled with a ventilation/perfusion mismatch, especially in otherwise healthy subjects. This observation is sustained by the small increase in partial arterial pressure of oxygen (PaO_2_) despite delivering increasing inspired oxygen fraction (FiO_2_) percentages, as it is frequently seen in COVID-19 patients during conventional O_2_ supplementation. Moreover, lung infiltrates appear to cause an “atypical” form of ARDS, in which an impressive shunt fraction (mean ± standard deviation; 0.50 ± 0.11) [4] is associated with a relatively high compliance (50.2 ± 14.3 mL/cmH_2_O) [4]. This event may be justified by the loss of lung perfusion regulation and hypoxic vasoconstriction [4], and by a hypercoagulability state [5] with consequent pulmonary microvascular coagulation. Post-mortem lung biopsies of COVID-19 patients demonstrated diffuse alveolar damage with cellular fibromyxoid exudates, interstitial lymphocytic infiltrates, desquamation of pneumocytes, and hyaline membrane formation [6]. This lung tissue damage results in a severe acute HRF characterized by significant hypoxia and normal or reduced partial pressure of carbon dioxide (CO_2_).

## 3. Rationale for Helmet Continuous Positive Airway Pressure (CPAP) in Severe COVID-19 Pneumonia

The application of positive end expiratory pressure (PEEP) during acute HRF secondary to pulmonary edema, atelectasis, or pneumonia has been demonstrated to improve arterial oxygenation by increasing functional residual capacity, shifting the tidal volume to a more compliant part of the pressure-volume curve, thus reducing both the work of breathing and the risk of tidal opening and closure of the airways [7]. Moreover, the application of PEEP recruits non-aerated alveoli in dependent pulmonary regions, stabilizes the airways, and reduces the inhomogeneity of lung volume distribution [7]. PEEP can be applied to spontaneous breathing patients in the form of Continuous Positive Airway Pressure (CPAP) [8]. CPAP is considered to be a valuable initial approach for patients suffering from acute de novo HRF and mild-moderate ARDS. In fact, non-invasive ventilation with a face mask can fail because of poor patient compliance and technical problems tied to the interface seal [9]. Around twenty years ago, the helmet had been proposed as an alternative to traditional interfaces. The helmet equipment, although slightly varying in some details between manufacturers, is schematically presented in Figure S1. Briefly, it consists in a transparent, latex free, polyvinylchloride hood joined by a metal or plastic ring to a soft polyvinylchloride collar of different sizes [10]. Two underarm straps are attached to the ring which keeps it from flying upwards when the gas flow pressurizes it [10]. The average volume of the hood ranges from 12 to 15 L with the patient’s head in place. The high flow gas enters the hood from one side, while on the opposite side, an expiratory port with an integrated manometer and an adjustable or fixed PEEP valve are applied (Figure S1). The anti-suffocation valve is applied on the hood’s surface and fixed by means of a screw shaped mechanism for rapid access to the inner part of the helmet (Figure S1).

Physiological studies demonstrated that the helmet and the face mask performed equally in reducing the inspiratory work of breathing during continuous high flow CPAP [10]. The non-invasive application of CPAP with the helmet significantly improved arterial oxygenation compared with standard oxygen therapy in patients with community acquired pneumonia [11]. Moreover, the helmet CPAP applied in patients with severe HRF due to pneumonia demonstrated to reduce the risk of meeting the criteria for endotracheal intubation compared with the Venturi mask [12]. Some data also suggest that the helmet CPAP decreased the incidence of endotracheal intubation in patients with severe acute HRF after abdominal surgery and in immunocompromised patients with hematological malignancy [13,14].

In a recent overview of the indications for the protection of healthcare workers from SARS-CoV-2 infection, Ferioli and coworkers showed how the helmets provided with a tight air cushion around the neck–helmet interface, in a double limb circuit, have negligible air dispersion during NIV application [15], and represent, together with the CPAP via oronasal mask, the ventilatory support that allows the minimum room air contamination [15]. In the current restricted availability of negative pressure rooms, we suggest applying an anti-viral filter both on the inspiratory and on the expiratory ports of the helmet (Figure S1). This should maximally reduce the risk of droplets dispersion. It should be noted that in case of sneezing or coughing, the helmet appears to be more practical and comfortable compared with conventional oronasal masks.

The helmet is generally more tolerated compared with the face mask, especially if CPAP therapy must be extended for several days, as it increases the comfort for the patients, reduces the risk of facial decubitus, allows patients to be fed and hydrated orally, and therapy to be administered without removing the helmet [10,16,17,18]. Finally, the helmet only needs an access to a high flow oxygen source (or a combination of compressed air and oxygen) without necessitating electricity.

## 4. CPAP Side Effects and Helmet Pitfalls

The administration of CPAP is not free from pitfalls. As it may cause over-distention of normal alveolar spaces causing barotrauma, it may also increase physiological dead space and reduce tissue perfusion [7]. Moreover, excessive PEEP may have detrimental effects on neuro-diaphragmatic coupling [19] and cardiac output, particularly in patients with preserved left ventricular function [7]. Finally, PEEP is often used to offset hyperinflation and intrinsic PEEP in patients with Chronic Obstructive Pulmonary Disease (COPD) during NIMV, but the prevalence of chronic pulmonary comorbidities in COVID-19 patients reported in literature so far was low (e.g., COPD ranging from 1.1% [20] to 10% [21]), leaving the need for NIV to a limited number of patients [18]. 

Compared with the face mask, the helmet, due to its larger internal volume, might facilitate CO_2_ rebreathing [22]. The inspired CO_2_ concentration was found to be constantly higher when CPAP was delivered by means of a continuous flow CPAP helmet (for flows ranging from 20 to 60 L and PEEP from 0 to 15 cmH_2_O) than with a face mask (mean ± standard deviation; 3.1 ± 0.15 versus 0.8 ± 0.3 mmHg, *p* < 0.01) [18]. This was also true when the helmet was tested during pressure support ventilation [23]. However, for CPAP delivery, higher flow rates corresponded lower inspired concentrations of CO_2_ [18]. The CO_2_ rebreathing depends primarily on two factors: (a) the fresh gas passing through the helmet, and (b) the amount of CO_2_ produced by the patient [22,24]. The presence of an antisuffocation valve (Figure S1) limits the CO_2_ rebreathing, but cannot prevent the loss of PEEP in case of gas flow interruption [22]. In this case, the adoption of larger valves allows for a lower CO_2_ rebreathing, but also a higher reduction in FiO_2_ [25]. When CPAP is administered by means of mechanical ventilators, it is generally not advisable to use the helmet [8,22] because the higher compliance of the helmet may cause a delay between the delivered inspiratory flow and patients’ inspiratory effort, which can cause patient-ventilatory asynchrony. Moreover, the mixing between the inspired and expired flows predisposes to CO_2_ rebreathing [8]. The most effective CPAP is achieved when the PEEP level is maintained throughout the respiratory cycle, with inspiratory fluctuations in PEEP reflecting an insufficient gas delivery compared with the patient minute ventilation [10]. This is why high flow systems should be preferred when administering CPAP with the helmet.

Taking into account the aforementioned limitations, especially the accidental gas flow interruption with a subsequent possible dangerous risk in PEEP and oxygen reduction, we underline that the application of the helmet CPAP should be always supported by appropriate and dedicated monitoring and alarming systems.

## 5. A Management Algorithm for COVID-19 Patients with De Novo Respiratory Failure

**Figure 1:**
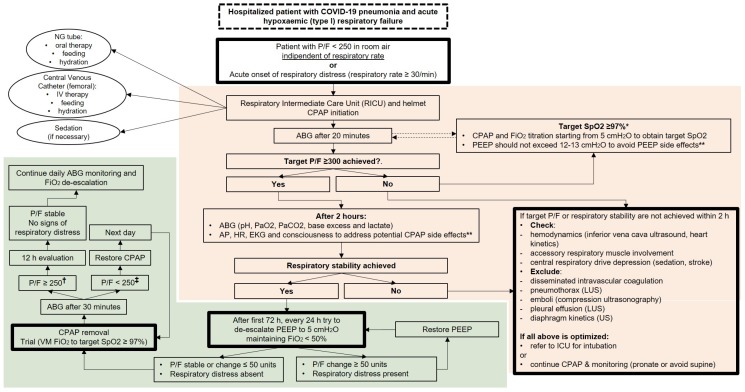
Decisional and monitoring algorithm for continuous positive airway pressure (CPAP) treatment in COVID-19 patients. Flow chart showing a decisional and monitoring algorithm for initiation, titration (red shadowed area), and de-escalation (green shadowed area) of the respiratory support with helmet continuous positive airway pressure (CPAP) in patients admitted with acute hypoxemic respiratory failure secondary to COVID-19 pneumonia. * If patient has metabolic alkalosis. If pH is within physiological limits, SpO_2_ target ≥95%. ** Cardiac output, O_2_ delivery, hyperoxygenation atelectasis, exacerbation of self induced lung injury. Patients at high risk of developing hypercapnic respiratory failure (COPD, emphysema, NMD) should be treated with NIV. ^†^ P/F ≥ 200 if patient has COPD or ≥70 years old; ^‡^ P/F < 200 if patient has COPD or ≥70 years old. P/F = partial pressure of oxygen to inspired oxygen fraction ratio; ABG = arterial blood gas analysis; AP = arterial pressure; EKG = electrocardiogram; FiO_2_ = inspired oxygen fraction; HR = heart rate; ICU = intensive care unit; IV = in vein; LUS = lung ultrasound; NG = nasogastric; PEEP = positive end expiratory pressure; P/F = partial pressure of oxygen to inspired oxygen fraction ratio; US = ultrasound; VM = Venturi Mask; PaO_2_ = partial arterial pressure of oxygen; SpO_2_ = peripheral O_2_ saturation; COPD = Chronic Obstructive Pulmonary Disease; PaCO_2_ = partial arterial pressure of carbon dioxide; NMD = neuromuscular disease; NIV = non-invasive ventilation.

Helmet CPAP is currently being extensively used in Italy during the COVID-19 pandemic. Despite the relative simplicity of setting up a CPAP helmet, the need for attentive and careful monitoring of the respiratory and hemodynamic response to the application of PEEP should be part of the standard operating procedures of the unit. Based on the observation and care provided so far to more than 70 COVID-19 patients with HRF in our RICU, we propose that the initiation of the CPAP treatment should always depend upon the PaO_2_/FiO_2_ ratio rather than on peripheral O_2_ saturation (SpO_2_) or the respiratory rate (RR), as falsely high SpO_2_ and low RR may be often secondary to the frequent mixed or metabolic alkalosis experienced by COVID-19 patients due to dehydration, iatrogenic diarrhea, and hypoalbuminemia [21,26]. In fact, an increase of pH value causes a left shift of the oxygen–hemoglobin dissociation curve, while hypocapnia makes the alveolar ventilation-PaO_2_ relationship much steeper only at values of PaO_2_ much lower than those usually considered as the cutoff for respiratory failure [26]. In addition, in the early phases of a progressive HRF, especially in case of otherwise healthy young subjects, the respiratory rate might be a less reliable sign of hypoxia due to the ability of these subjects to increase the minute ventilation by increasing the tidal volume before showing signs of respiratory distress [27]. Therefore, arterial blood gas analysis should be used as the main monitoring tool for establishing the need for CPAP and its titration. Initiating CPAP with pressures as low as 5 cmH_2_O should allow to control and adjust for potential cardiovascular and pulmonary side effects (Figure 1). In our opinion, PEEP should not exceed 12–13 cmH_2_O in order to avoid barotrauma, self-induced lung injury, tension pneumothorax, and negative effects on hemodynamics [7]. Moreover, it has been suggested that COVID-19 pulmonary infiltrates are associated with poor recruitability, thus high PEEP should be avoided to preserve patients from severe hemodynamic impairment and fluid retention [4]. Coppola and coworkers [28] have recently demonstrated that in patients with ARDS undergoing lung protective ventilation, the amount of intrinsic PEEP is negligible. If present, intrinsic PEEP did not change the total airway resistance, compliance of the respiratory system, and lung recruitability [28]. This observation, along with the aforementioned PEEP side effects [7], sustains the hypothesis that higher levels of PEEP (e.g., >15 cmH_2_O) during non-invasive CPAP application and without the continuous monitoring of lung mechanics available only in the ICU should be avoided.

Currently, to our knowledge, there are no shared recommendations on weaning from CPAP in adults with HRF and severe pneumonia [29], and so far, the majority of evidence on this topic comes from pediatric/neonatal studies [30]. We propose that, as the patient reaches clinical and respiratory stability, the weaning from the helmet should start from reducing the PEEP to the lower possible value (for the helmet, usually 5–6 cmH_2_O) maintaining a FiO_2_ not higher than 50%. If lung derecruitment is absent and the P/F ratio is stable as compared with higher PEEP values (Figure 1), the patient is ready to undergo a CPAP weaning trial. A weaning trial should be attempted every day to avoid a delay in CPAP removal.

We are aware that the latter approach is based on clinical observation and not on data from randomized clinical trials, but we believe that this procedure may reduce the number of patients that fail weaning from CPAP and expedite the course of hospitalization.

During the writing of the present manuscript, the Surviving Sepsis Campaign (SSC) has released new guidelines for the management of critically ill adults with COVID-19 [31]. The authors recommend the use of high flow oxygen therapy and non-invasive positive pressure ventilation if high flow oxygen is unavailable or ineffective in COVID-19 patients with acute respiratory failure [31]. Surprisingly, the application of CPAP is not mentioned [31]. Due to the pathophysiology of the acute respiratory failure in COVID-19 patients, we believe that an approach based on non-invasive ventilation, unless hypercapnia is present, could be avoided. Considering also the poor availability of negative pressure rooms in the majority of hospitals involved in the current pandemic, and the need for preserving the healthcare personnel from viral contamination, an approach based on non-invasive ventilation and high flow oxygen therapy should be left only to specific and protected settings [15,32]. In Table S1, we present preliminary data about in hospital mortality of patients treated in our RICU, compared with data available in literature so far. Although care settings and populations are inhomogeneous, in the majority of cases, the ARDS severity and mortality of patients treated with the helmet CPAP is comparable to ICU cohorts (Table S1).

## 6. Conclusions

The complexity of the clinical picture in patients with COVID-19 related HRF deserves great attention in the identification of patients with high risk of rapid respiratory function deterioration. The application of CPAP with the helmet can represent a valid pulmonary support in the adequate setting and with simple monitoring tools. A careful CPAP titration can optimize the recruitment of unventilated lung regions and improve hypoxemia, making it a suitable bridge to ICU or a supportive treatment to improve patients’ outcomes. A better tolerability of the helmet and a reduced room contamination compared with oronasal masks may also improve patients’ clinical management, increasing the safety of the healthcare workers involved in the assistance during the COVID-19 pandemic.

## Supplementary Materials

The following are available online at https://www.mdpi.com/2077-0383/9/4/1191/s1, Figure S1: Schematic representation of the helmet CPAP equipment, Table S1: COVID-19 patients’ severity and in-hospital mortality rate for studies published until 8 April 2020. References [33,34,35,36,37,38,39] are cited in Supplementary Materials.
