# Supplementary material for: Helmet CPAP to Treat Acute Hypoxemic Respiratory Failure in Patients with COVID-19: A Management Strategy Proposal
Source: J Clin Med. 2020 Apr 22;9(4):1191. doi: 10.3390/jcm9041191 (PMC7230457; doi:10.3390/jcm9041191)
Supplement: Supplementary file 1 [file jcm-09-01191-s001.pdf]

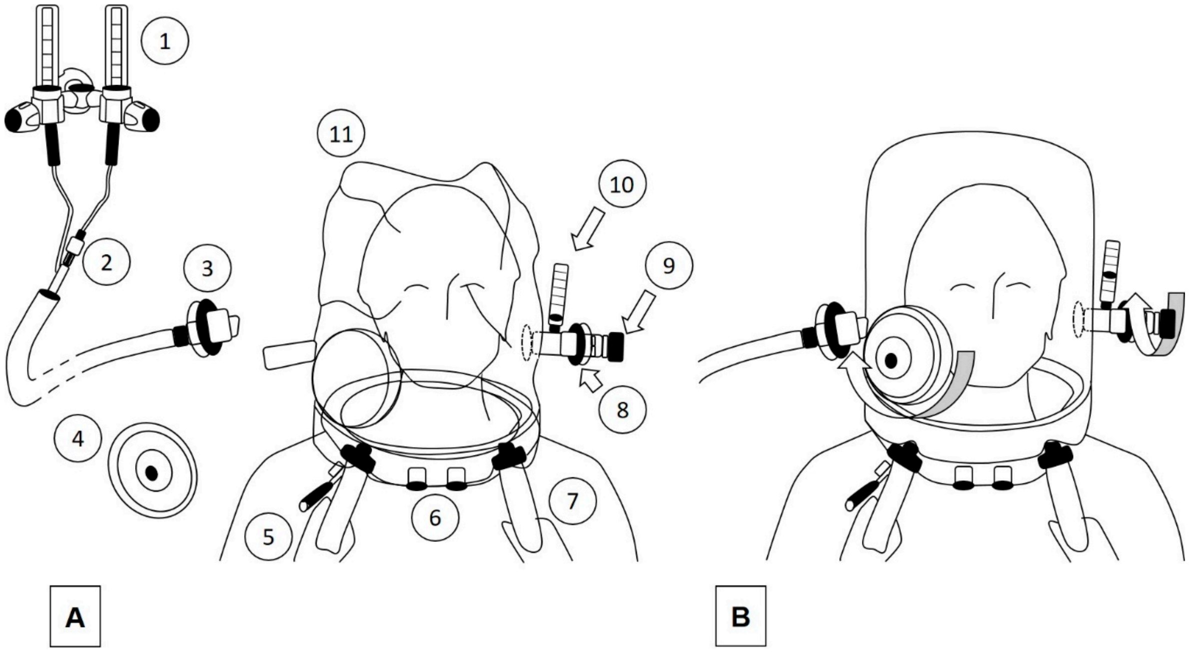

Figure S1. Schematic representation of the helmet CPAP equipment. (**A**) shows the mounted deflated helmet just before being connected to the high flow oxygen circuit. When connected to gas flow (**B**) the antisuffocation valve must be in place and the helmet must remain inflated during inspiratory efforts. 1 = double oxygen flow meter (maximal flows of 5 and 30 L, respectively). The right combination of flows to obtain the desired FiO_2 depending on the PEEP setting is usually reported in a leaflet and depends on the available high-flow mechanism (e.g., air + oxygen blender). 2 = venturimeter valve must be closed to obtain 100% FiO_2. 3 = anti-bacterial filter. 4 = antisuffocation valve (compare with (B); valve in place. The curved arrow indicates a screw like mechanism); 5 = inflatable air pillow that runs around patients' neck for improved comfort and air seal; 6 = small additional operational ports to allow the passage of the nasal gastric tube or for maintaining the seal and reduce contamination while the patient drinks by means of a straw; 7 = armpit straps for anchoring the helmet; 8 = 100% anti-viral and anti-bacterial filter; 9 = PEEP valve, which can be regulated with a spring mechanism (see also the curved arrow in (B)); 10 = manometer column, to have a direct reading of the real-time pressure changes inside the helmet; depending on the helmet design, it can be replaced by spot reading by means of a hend-held manometer through a small port usually placed on the filter crown; 11 = plastic hood (deflated). CPAP = continuous positive airway pressure; FiO_2 = inspired oxygen fraction; PEEP = positive end-expiratory pressure.

Table S1. COVID-19 patients' severity and in-hospital mortality rate for studies published until 8 April 2020.

Region	Setting	Median (IQR) P/F #	Respiratory Support	In-Hospital Mortality
30 provinces, China [21]	ICU and other	225 (158–293) * severe patients	46.9% MV (32.4% NIV–14.5% IMV)	8.1%
		165 (120–304) * composite outcome **	80.6% MV (43.3% NIV–37.3% IMV)	22.4%
Wuhan, China [33]	ICU and other	54% of patients with P/F ≤ 300<br>31% of patients with P/F ≤ 200	21% HFNC; 31% MV (14% NIMV–17% IMV)	28.3% (80.7% †)
Wuhan, China [34]	ICU and other	76% of patients with P/F ≤ 300	20% MV (12.3% NIV–7.7% IMV)	18%
Wuhan, China [35]	ICU	198 (147–255)	30.1% HFNC; 30.2% MV (11% NIV–19.2% IMV)	28.8%
Wuhan, China [36]	ICU	279 (157–328)	10.2% HFNC; 40% MV (9.9% NIV–29.1% IMV)	38.7%
Lombardy, Italy [37]	ICU	160 (114–220)	99% MV (11% NIV–88% IMV)	26%
Seattle area, WA (US) [38]	ICU	142 (94–177) ‡	100% IMV	50%
Division or Respiratory Diseases, "L. Sacco" University Hospital, Milano, Italy	RICU	138 (87–267)	73% Helmet CPAP	24%

ICU = intensive care unit; RICU = respiratory intermediate care unit; P/F = PaO_2/FiO_2 ratio; IQR = inter quartile range; MV = mechanical ventilation; IMV = invasive mechanical ventilation; NIV = non-invasive mechanical ventilation; HFNC = high flow nasal cannula; CPAP = continuous positive airway pressure. The study by Cao et al. [39] was excluded from the present report because among the 58 patients with ARDS none was deceased at the time of the publication. # when available; * missing for 81,3% of patients. Converted in mmHg from kPa; ** including death, ICU or need for invasive mechanical ventilation; † among patients with respiratory failure; ‡ at day 1 of mechanical ventilation.
